# Increased resistin in brain dead organ donors is associated with delayed graft function after kidney transplantation

**DOI:** 10.1186/1479-5876-11-233

**Published:** 2013-09-26

**Authors:** Simona Oltean, Rille Pullerits, Anne Flodén, Michael Olausson, Mihai Oltean

**Affiliations:** 1 The Transplant Institute, Sahlgrenska University Hospital, Gothenburg 41345, Sweden; 2 Department of Clinical Immunology and Transfusion Medicine, Sahlgrenska University Hospital, Gothenburg, Sweden; 3 Department of Infectious Diseases, University of Medicine and Pharmacy, Cluj-Napoca, Romania; 4 Department of Rheumatology and Inflammation Research, Institute of Medicine, The Sahlgrenska Academy at University of Gothenburg, Gothenburg, Sweden; 5 Organ Donation Unit, Sahlgrenska University Hospital, Gothenburg, Sweden; 6 Department of Surgery, Institute for Clinical Sciences, The Sahlgrenska Academy at University of Gothenburg, Gothenburg, Sweden

## Abstract

**Introduction:**

Resistin increases during several inflammatory diseases and after intracerebral bleeding or head trauma. Resistin activates the endothelium and may initiate an inflammatory response. No data are available on resistin in brain dead donors (DBD) that regularly manifest a pronounced inflammatory state.

**Methods:**

We analyzed plasma resistin in 63 DBDs and correlated results with donor variables and the postoperative course following kidney transplantation using organs from these donors. Endocan and monocyte chemotactic protein (MCP)-1 were also studied. Twenty-six live kidney donors (LD) and the corresponding kidney transplantations were used as controls.

**Results:**

DBDs had higher resistin (median/range 30.75 ng/ml, 5.41–173.6) than LD (7.71 ng/ml, 2.41–15.74, p < 0.0001). Resistin in DBD correlated with delayed graft function (DGF) in the kidney recipients (r = 0.321, p < 0.01); receiver operating characteristic curve revealed an area under the curve of 0.765 (95% confidence interval [CI] 0.648–0.881, p < 0.01) and a cut-off value for resistin of 25 ng/ml; MCP-1 and endocan were higher in DBDs (p < 0.0001) but did not correlate with DGF or acute rejection. No relationship was found between the studied molecules and the postoperative course of LD kidney transplants.

**Conclusions:**

High resistin levels in the DBD before organ retrieval are associated with DGF after kidney transplantation. The resistin increase seems related to the inflammatory state after brain death but not to the cause of death.

## Introduction

Brain death triggers a complex cascade of molecular and cellular events including the release of various pro-inflammatory mediators and leading to a pronounced inflammatory state. The triggering stimulus of this phenomenon remains unknown, but it eventually results in endothelial and complement activation, massive cytokine release, hemodynamic impairment and ultimately an immunologically activated organ before transplantation [1-4]. These changes increase the susceptibility for both ischemia-reperfusion injury as well as rejection, and may provide an explanation for the inferior results following transplantation of organs from deceased donors as compared with living donors [5].

Resistin has been initially described as an adipokine related to the insulin resistance in obese mice. In humans, the expression pattern of resistin is different [6] and human resistin, synthesized predominantly by mononuclear cells, has features similar to classical pro-inflammatory cytokines playing a role in inflammation and immunity [7,8]. The recent experimental and clinical evidence has revealed a possible role of resistin in diverse pathological settings such as atherosclerosis [9], rheumatic diseases [10], cancer [11] and several other diseases [12-14]. Increased resistin levels have been reported during these inflammatory conditions. It has been demonstrated that pro-inflammatory cytokines can induce strong upregulation of resistin in peripheral blood mononuclear cells [8,15] and in turn, resistin itself can promote inflammation through induction of a cytokine cascade [8,16]. The systemic resistin increase seems to be related with the active disease and the extent of organ injury or dysfunction [13,14,17,18]. To date, very little is known about the role of resistin after organ transplantation and there are no published data available on resistin in organ donors.

Resistin is able to promote the endothelial cell activation and mount a robust pro-inflammatory response [19]. Thus, resistin may represent an injury marker as well as a pro-inflammatory signal, which contributes to the inflammatory cascade after brain death. Other established markers of endothelial activation and injury include several soluble cell adhesion molecules, chemokines or other endothelium-derived biomolecules (i.e., von Willebrand factor, glycoproteins, proteoglycans). Endocan is a proteoglycan expressed by the endothelial cells that binds to human leukocytes via the integrin leukocyte function-associated antigen (LFA)-1 and can also be detected free in the blood [20]. Inflammatory cytokines induce an up-regulation of endocan messenger RNA and the release of the molecule by the endothelium [20]. Endocan has recently been suggested as a novel endothelial dysfunction marker with a higher discriminative value for predicting septic shock and death than the von Willebrand factor [20].

In this study we analyzed the circulating levels of resistin in brain dead organ donors and in healthy living donors at the time of organ procurement and studied its relationship with two markers of endothelial activation such as endocan, and monocyte chemotactic protein (MCP-1) as well as the early post-transplant course.

## Materials and methods

### Patients and samples

Plasma samples were obtained from 63 deceased brain dead (DBD) multiorgan donors from our procurement area between August 2006 and March 2012. The donors (or next of kin) previously consented for blood/tissue donation for the purpose of medical research. Individuals donating a kidney for transplantation i.e. living donors (LD) at our unit served as healthy controls (n = 26). The study was approved by the Ethical Committee of Gothenburg University and consent was obtained from all individuals.

Blood was drawn on EDTA-tubes from DBD donors just prior to the organ recovery procedure. Following centrifugation plasma was recovered, aliquoted and stored at −80°C until analysis. Donor information regarding age, gender, cause of death (COD), steroid pretreatment, donors’ last creatinine, C-reactive protein and body mass index (BMI) were retrieved. Plasma samples were also obtained from healthy kidney donors and prepared as above.

### The early outcome after kidney transplantation

The early behavior of the kidneys retrieved from the group of above described donors and transplanted in adult recipients at our center was followed. Five kidneys were discarded before transplantation due to underlying pathology (cysts, sclerosis). The kidneys shipped to other Nordic transplant centers according to Scandiatransplant exchange rules (n = 20), those transplanted together with the liver (n = 9), or early technical failure (n = 1) were excluded from the analysis.

The medical records of the remaining 91 kidney graft recipients were reviewed and information retrieved regarding cold ischemia time, recipient demographics, graft function (creatinine), need for hemodialysis during the first week after transplantation and occurrence of rejection during the first month. Day of surgery (release of vascular clamps) was considered day 0. All kidneys underwent static cold preservation in either University of Wisconsin or histidine-tryptophane-ketoglutarate solution. Kidneys from living donors were transplanted within an hour from donor nephrectomy. Recipient immunosuppression was based on antibody induction therapy (basiliximab or rituximab), calcineurin inhibitors, mycophenolate mofetil and steroids.

The primary endpoint of the study was delayed graft function (DGF), defined as requirement for hemodialysis during the first week after transplantation. A secondary endpoint was slow graft function (SGF) not requiring dialysis, defined by a decrease in recipient serum creatinine <30% from posttransplant day 1 to day 2 [21,22]. Creatinine reduction rate at day 2 (CRR2) was calculated using the formula:

CRR2 (%): (Creatinine Day 1-Creatinine Day 2) * 100/Creatinine Day 1.

### Measurement of resistin, MCP-1 and endocan

Resistin was measured using a colorimetric sandwich ELISA kit (DRSN00); MCP-1 was measured using a Duo Set ELISA development kit (both from RND Systems, Minneapolis, MN) following manufacturer’s instructions. The lower detection limits were 0.16 ng/ml for resistin and 15.62 pg/ml for MCP-1.

Endocan is a proteoglycan expressed only by the endothelium, constitutively present on several vascular beds including the glomerular and peritubular capillaries [23]. Endocan has been used as surrogate marker of endothelial injury and activation and measured in the plasma of deceased and living organ donors using a Human endocan/ESM-1 DIY ELISA Kit (LIK-1101, Lunginnov, Lille, France). The lower detection limit of the assay was 0,16 ng/ml.

### Statistical analyses

Following the analysis of data distribution, Student’s *t*-test and analysis of variance were used to calculate samples with normal distribution, whereas the Kruskal-Wallis test and Mann–Whitney *U* test were used for the analysis of the nonparametric data. Fisher’s exact test was employed for analyses of contingency tables. The parametric correlations were assessed using the Pearson correlation coefficient, and the nonparametric correlations were assessed using the Spearman correlation coefficient. Receiver operating characteristic curve (ROC) analysis was performed to assess the potential of resistin to predict DGF. Data are expressed as median and range, unless otherwise stated. A p-value < 0.05 was considered significant.

## Results

### Characteristics of donors and recipients

Table 1 summarizes donor (n = 63) and recipient (n = 91) characteristics in the group of DBD kidney transplantations, while Table 2 shows the details of the living donor (LD) kidney transplantations (n = 26).

**Table 1:** Characteristics of the deceased brain-dead donors and corresponding kidney recipients

***Donors (n = 63)***	**n (%)**
Mean age, years (± SD)	51.6 (± 15.6)
Male	36 (57.2%)
Cause of death, n (%)	
Cerebrovascular accident	45 (71.4%)
Traumatic brain injury	10 (15.8%)
Hypoxic brain damage	6 (9.6%)
Other	2 (3.2%)
Mean ICU days (± SD)	2,28 (± 2)
Inotropes	50 (79.3%)
Steroid pretreatment	38 (60%)
History of hypertension	18 (28.57%)
Mean BMI (± SD)	25.17 (± 4.66)
Mean plasma creatinine, μmol/L (± SD)	82.82 (± 42.78)
Mean eGFR, mL/min (± SD)	96.15 (± 43.35)
ECD	26 (41.26%)
***Recipients (n = 91)***	
Mean age, years (± SD)	52.3 (12.44%)
Male, n (%)	56 (61.53%)
Polycystic disease	14 (15.3%)
Glomerulonephritis	39 (42.9%)
Diabetes mellitus	18 (19.9%)
Other	20 (21.9%)
First transplantation	67 (73.6%)
Retransplantation	24 (26.7%)
Antigen mismatches-A, B and DR (mean ± SD)	3,7 ± 1,3
Mean cold ischemia time, min (± SD)	802 (± 295.3)
SGF Slow graft function	73 (80,2%)
Delayed graft function	13 (14.3%)
Acute rejection during the first month	6 (6.6%)

*Abbreviations:eGFR* estimated glomerular filtration rate using the modification of diet in renal disease (MDRD) equation for glomerular filtration rate in the 61 adult donors and the Schwartz equation in two donors under 18 years of age, *SD* standard deviation, *ECD* extended criteria donors include any donor over 60 years old and donors 50–59 years old with at least two of the following: terminal creatinine >1.5 mg/dL (114 μmol/ml), history of hypertension, or death by cerebrovascular accident, *DGF* delayed graft function, *SGF* Slow graft function, < 30% creatinine reduction rate at day 2.

**Table 2:** The living donor kidney transplantations

**Living donors (n = 26)**	
Mean age, years (± SD)	48.2 (± 13.7)
Male	8 (30.7%)
Mean body mass index	25.52 (± 4.66)
Mean plasma creatinine, μmol/L (± SD)	82.82 (± 42,78)
Mean eGFR, mL/min (± SD)	116 (± 34.8)
Recipients (n = 26)	
Mean age, years (± SD)	45 (±12,44)
Male, n (%)	18 (69.2%)
Polycystic disease	3 (11.5%)
Glomerulonephritis	10 (42.4%)
Diabetes mellitus	4 (15.4%)
Other	9 (30.7%)
First transplantation	22 (84.6%)
Retransplantation	4 (15.4%)
Antigen mismatches-A, B and DR (mean ± SD)	2,8 ± 1,8
Delayed graft function	0
Acute rejection during the first month	8 (30.7%)

Characteristics of the living kidney donors and kidney recipients. Donor GFR has been measured directly using either Cr-EDTA or Iohexol clearance.

After transplantation, delayed graft failure (DGF) occurred in 13 (14.28%) of DBD kidney recipients, whereas none of the LD kidney recipients developed DGF. Slow graft function (SGF) was observed in 73 (80.2 %) of DBD kidney recipients and in 10 (38.4%) recipients of LD.

### Donor resistin and the post-transplant course

**Figure 1:**
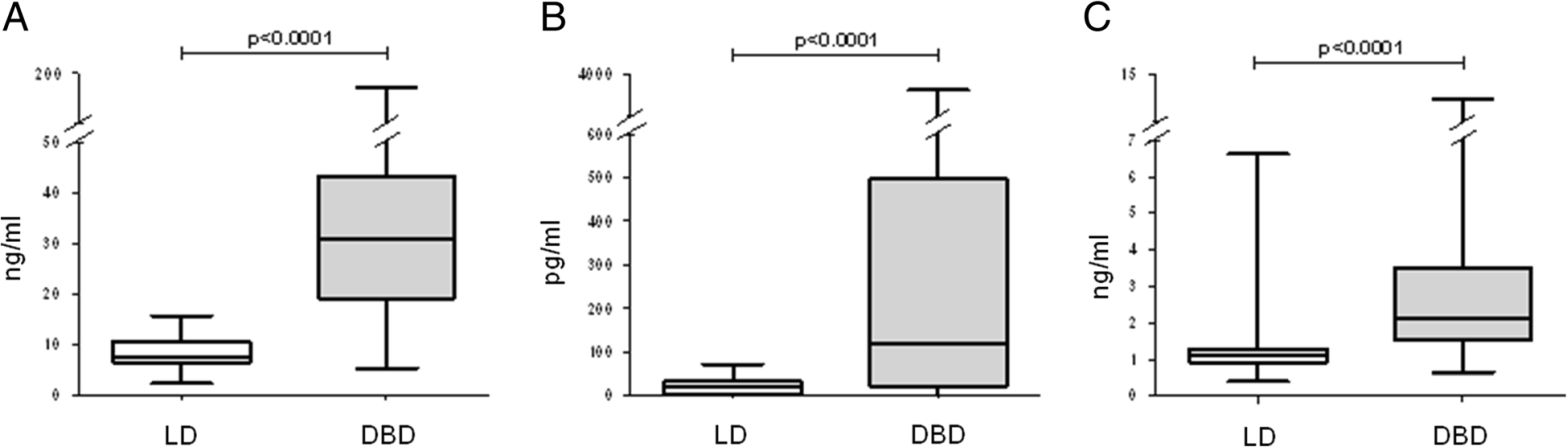
**The enzyme-linked immunosorbent assay (ELISA) results in the plasma of brain dead organ donors (DBD, grey box) and living donors (LD, open box).** DBDs had higher plasma concentrations of resistin **(A)**, MCP-1 **(B)** and endocan **(C)** as compared with LD.

Resistin levels were significantly higher in the DBD and had a median value of 30.75 ng/ml (5.41–173.6) compared with a median of 7.71 ng/ml (2.41–15.74) the in LD (p < 0.0001) (Figure 1A). Resistin concentration was not influenced by the cause of death (Table 3) or the donor body mass index (BMI). Steroid pretreated donors had resistin levels not significantly different from untreated DBDs.

**Table 3:** Median (range) plasma resistin, MCP-1 and endocan concentrations in the brain dead donors according to the cause of death

	**Resistin (ng/ml)**	**MCP-1 (pg/ml)**	**Endocan (ng/ml)**
Major vascular accident (n = 45)	28.7 (5.41–157)	121 (0–3723)	2.1 (0.654–7.87)
Trauma (n = 10)	34.3 (12.6–54.6)	122 (1.13–1800)	2.84 (1.13–3.92)
Hypoxia (n = 6)	34.3 (16.6–174)	190 (0–2675)	2.68 (1.17–12.4)
Other (n = 2)	71.73 (59.46–84)	275.7 (52.5–490)	1.56 (1.13–1.99)

*MVA* major vascular accident.

**Figure 2:**
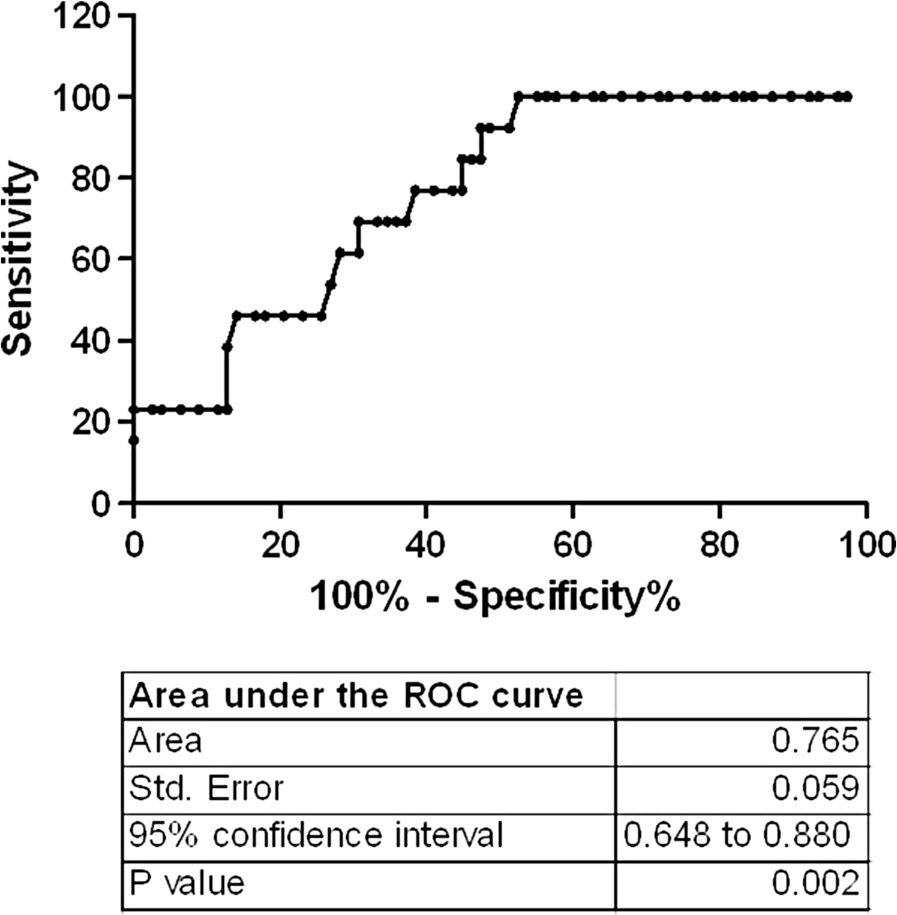
**Receiver operating characteristic curve of resistin as predictor of DGF.** Area under the curve followed by SE and 95% CI are shown.

When referring to DGF (need for hemodialysis during the first posttransplant week), resistin was significantly increased in the DBD donors of kidneys that required dialysis compared with the donors of kidneys not requiring dialysis: 41.87 (26.47–173.6) ng/ml vs.27.24 (5.41–149.3) ng/ml, p < 0.01. Resistin levels in the donor correlated moderately with recipients’ need for dialysis during the first week after transplantation (r = 0.321, p < 0.01). ROC curve analysis for resistin differentiating between recipients requiring HD (n = 13) and those not requiring (n = 78) hemodialysis gave an area under the curve (AUC) of 0.765 (95% confidence interval 0.648–0.881, p < 0.01) (Figure 2). The cutoff value for resistin in predicting DGF as revealed by the ROC curve was 25.

The association found by Fisher’s exact test between the plasma resistin levels above 25 mg/ml in DBDs and the risk of DGF is shown by an OR of 24,4 (95% CI 1,4–425, p < 0.001). The calculated positive predictive value for concentrations above 25 ng/ml was 0.241 while the negative predictive value was 1.

While grouping the donors according to the resistin levels, donor age did not differ between (51,5 ± 14 vs. 51,5 ± 16 years, p = 0.7). Donors with resistin less than 25 ng/ml had lower last creatinine (66,9 ± 24 vs. 95,3 ± 47, p < 0.01) and a higher estimated GFR (112 ± 40 vs. 85 ± 42 ml/min/1.73m^2^, p < 0,01) than the donors with resistin above 25 ng/ml. Vasoactive support has been used in 66% and 41% of the donors with low (<25 ng/ml) and high resistin (>25 ng/ml), respectively (p-ns.).

**Figure 3:**
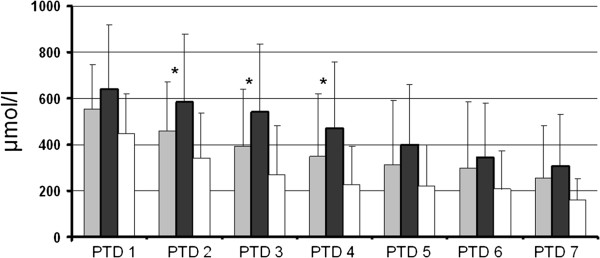
**The decrease in post-operative serum creatinine concentrations during the first week in recipients of kidneys from DBD donors with resistin <25 ng/ml (grey bars, n = 24), resistin >25 ng/ml (black bars, n = 39) and living donors (white bars, n = 26).** * p < 0.05.

The average creatinine between days two and four was significantly higher in recipients of kidneys from donors with resistin >25 ng/ml (Figure 3). The difference disappeared by day five possibly due to the initiation of renal replacement therapy in the kidney recipients showing inadequate graft function.

Further correlations between resistin and other donor-related variables and post-transplant events are presented in Table 4.

**Table 4:** Correlation matrix (Spearman’s rank) for several inflammatory biomarkers and renal function in the donor and post-transplantation events with serum resistin levels

	**DBD**	**LD**
**Markers of inflammation in the donor**		
CRP	0,246 (0,062)	-
Endocan	0,394 (0,001)	−0.115 (0.575)
MCP-1	0,353 (0,005)	−0.191 (0.35)
**Renal function in the donor**		
Last Creatinine	0,286 (0,025)	-
eGRF	−0,351 (0,006)	0.055 (0.791)
**Clinical events after transplantation**		
DGF	0.321 (0.002)	0
SGF	0.113 (0.287)	0.2 (0.327)
AR	0.133 (0.208)	0.1 (0.627)

The associations between resistin levels and inflammatory biomarkers, parameters of renal function as well as post-transplantation events in the deceased brain-dead (DBD) and living (LD) donors. Spearman’s correlation coefficients (p value) are shown.

BMI-body mass index, LOS-length of stay, AR-acute rejection, DGF-delayed graft function.

### Donor endothelial activation and the post-transplant course

The median MCP-1 level in the DBD was 1118 pg/ml (0.4–6624) whereas the median MCP-1 level in LD was significantly lower: 20.21 pg/ml (0–72.47) (p < 0.0001) (Figure 1B). Steroid pretreated donors had significantly lower MCP-1 concentrations: 44.93 pg/ml (0–3723) vs. 506.8 pg/ml (14–2675) (p < 0.0001).

Endocan levels were found significantly increased in DBD compared with LD: 2.14 ng/ml (0.65–12.36) vs. 1.12 ng/ml (0.41–6.62) (p < 0.0001) (Figure 1C). Donor COD or steroid pretreatment did not influence endocan concentrations. Both MCP-1 and endocan levels correlated moderately with resistin concentrations (Table 4).

## Discussion

Brain death induces an intense pro-inflammatory state through several, incompletely known mechanisms. This increases graft susceptibility to ischemia-reperfusion injury but it may also cause direct injury to various transplantable organs, thus explaining the inferior results after transplantation of organs from deceased brain dead donors [3,5,24]. In the present study we found significantly higher concentrations of resistin in brain dead organ donors and report for the first time an association between increased resistin levels and the development of DGF in the early posttransplant course.

The endothelium is a prime site of the brain death-induced organ injury [25]. Endothelial cells are resistin-sensitive cells, responding to resistin with up-regulation of vascular cell adhesion molecules and MCP-1 [19]. Previous studies have shown sizeable MCP-1 increase in brain dead donors and identified elevated donor MCP-1 as predictive for graft related complications after simultaneous pancreas-kidney transplantation [26] and continued MCP-1 release after lung transplantation has been related to unfavorable postoperative course [27]. We found that endothelial activation and endothelial injury in DBD donors are reflected by significantly increased MCP-1 and endocan levels. The moderate correlation between MCP-1 and resistin suggests that additional stimuli or mechanisms other that resistin may have contributed to the MCP-1 release.

Recent studies point out resistin as a ligand for Toll-like receptor 4 that mediates the pro-inflammatory effects of resistin in human cells [28]. Resistin can induce dose-dependent activation of mononuclear cells with intracellular signaling occurring through the NF-kappa B pathway, the same pathway that controls transcriptional activation and gene expression of major pro-inflammatory cytokines, chemokines and endothelial adhesion molecules involved in leukocyte adhesion and transmigration from blood vessels to interstitium [12,19]. Similarly, our study found that the early behavior of the kidney grafts depends on the concentrations of resistin in the donors. The distinct pattern of creatinine fall between kidneys coming from donors with low or high resistin may suggest that the difference is due to different degrees of ischemia-reperfusion injury but in the absence of biopsy findings this hypothesis remains speculative.

The association between resistin and MCP-1 in the brain dead organ donors seems to indicate a causative relationship between these two and may suggest a role for resistin in the initiation of the inflammatory response after brain-death. Unlike MCP-1, resistin did not appear influenced by the steroid pretreatment, suggesting either a different mechanism (i.e., passive release instead of de-novo synthesis) or a more upstream position in the inflammatory cascade that is not influenced by medication. The effect of steroid treatment on resistin and endocan-1 is more difficult to assess since our data come from only one, rather late time-point. This hypothesis should be preferably studied at several time points before and after the intervention.

Increased resistin concentrations were reported after head trauma or intracerebral bleeding [29-31]. Several studies suggest that resistin levels are proportional with the magnitude of injury and indicative for prognosis following ischemic or hemorrhagic stroke [29,31]. Interestingly, resistin concentrations observed after head trauma or intracerebral bleeding were similar with the concentrations reported herein. It seems unlikely that resistin leaked from the injured brain into the systemic circulation since some brain-dead individuals with severe brain infarction had only modest increases in resistin levels. Moreover, the missing cerebral circulation implies an absent venous drainage of the brain tissue. Although the exact source of resistin in DBD donors remains unclear, it is plausible that dysregulation of neural and hormonal signals occurring after donor brain death leads to the release of resistin from activated immune cells.

Increased resistin levels have been reported in patients with chronic renal disease and interesting relationships between resistin concentration, glomerular filtration rate and several biomarkers of inflammation have been identified [32,33]. Although the exact mechanisms are unclear, the study by Axelsson et al. [32] suggested that hyper-resistinemia appears to be linked to inflammation but not to obesity and insulin resistance. Comparable results were reported in a small cohort of patients with IgA nephritis [34]. Interestingly, our study found a statistically significant (albeit clinically marginal) difference in the renal function of donors with resistin above or below the cut-off value identified herein. Whether this is secondary to the inflammatory, brain death–induced kidney injury or due to preexisting renal impairment remains unclear. One interesting theory that arose from those studies is that resistin secretion through the kidneys may be the major pathway of its elimination. The inverse correlation between donor estimated GFR and resistin, further supports the hypothesis that increased resistin may be associated with renal damage.

Endocan is expressed only by the endothelium and constitutively present on several vascular beds including the glomerular and peritubular capillaries [23], although a circulating endocan fraction can also be normally detected. It has been suggested as a marker of endothelial dysfunction and increased endocan levels have been signaled in septic patients [20]. Limited information is available in critically ill patients and there is no published evidence on the role of endocan during aging or kidney diseases. The increased endocan concentrations in the brain dead donors and its correlation with MCP-1 may suggests that the increase in soluble endocan is part of the inflammatory response, but it remains unclear if this increase is indicative of organ injury. Further studies in patients with specific diseases could shed more light on the role of endocan as a marker of organ injury.

Donor age and the inotrope support are critical donor-related variables that may influence kidney graft function, yet several more subtle donor parameters such as the inflammatory milieu have been recognized to have impact on the transplant outcome [34]. Currently, the assessment of kidney function at the time of organ procurement is based mainly on routine parameters such as urine output or serum creatinine, although the later is a relatively insensitive marker of renal dysfunction and increases only in the late phases of injury. Additional information about donor and graft could assist in the initial decision making such as organ allocation or choice of immunosuppression. Several molecular changes in the organ donor have been suggested to correlate with early graft function and post-transplant outcome but have not yet been adopted into the clinical routine [22,35-38]. The significant correlation between the pro-inflammatory resistin and DGF and the cut-off value identified by our study as well as its rapid and simple assessment may suggest a place for resistin in a future panel of inflammation markers used in the assessment of the organ donor as well as a parameter with prognostic value for the function of the renal grafts.

Delayed graft function is unequivocally defined as the requirement for dialysis during the first post-transplant week, whereas slow graft function has several definitions and consequently, varying frequency [22,39-41]. The rate of DGF seen in our study is similar with that reported in the literature. However, the rather high threshold of 30% for creatinine reduction rate at day 2 we adopted could have contributed to a higher frequency of SGF. On the other hand, the high frequency could be attributable to donor-related factors since almost half of the transplanted kidneys came from extended criteria donors [42].

The present work is a single-center study and has the inherent limitations of any small series. In an attempt to ensure an overall good pre-transplant organ quality and facilitate the post-transplant comparisons we chose to include only multiorgan DBDs and this may have resulted in a selection bias. Also, the relatively low number of donors and kidney transplants prevented further in-depth subgroup analyses. However, our single center study had the advantage of managing both organ donors and transplant recipients according to similar protocols and routines throughout the study. Although non-consecutive, the donors in our study mirror well the donors at our center with respect to age, gender and cause of death.

The collection of data from only one time-point is another limitation of the study. This prevented detailed mechanistic studies but provided an identical, clinically feasible time-point. We are currently conducting a prospective study analyzing the time-course of resistin at several time-points between the declaration of brain death and organ retrieval.

## Conclusions

This first report investigating resistin in brain-dead organ donors indicates that high resistin levels in the donor at organ retrieval are associated with delayed graft function. The increase in resistin levels is not influenced by the cause of death but rather reflects the inflammatory state after brain death. Our observations need to be confirmed in larger donor and recipient series and should also include extended criteria donors, where any additional information could provide key data regarding organ allocation.

## Competing interests

The authors declare that they have no competing interests.

## Authors’ contributions

SO designed the study, collected and analyzed data, wrote the manuscript; RP analyzed data and wrote the manuscript; AF collected data and reviewed the manuscript; MOla reviewed data and manuscript; MOlt designed the study, collected and analyzed data and wrote the manuscript. All authors read and approved the final manuscript.
